# Supplementary material for: The RNA-binding activity of the Drosophila Brat protein is necessary for viability and mRNA regulation
Source: RNA Biol. 2026 May 28;23(1):1–19. doi: 10.1080/15476286.2026.2682069 (PMC13245067; doi:10.1080/15476286.2026.2682069)

**Supplementary Figures**

RNA-binding is the essential biological function of the *Drosophila* protein Brat

Robert P. Connacher^1^, Yichao Hu^2^, Richard Roden^1^, Julia Toledo^1^, Anna DesMarais^1^, Michael O’Connor^3^, Howard D. Lipshitz^2^, Aaron C. Goldstrohm^1^*

^1^ Department of Biochemistry, Molecular Biology, & Biophysics, University of Minnesota, USA

^2^ Department of Molecular Genetics, University of Toronto, Canada

^3^ Department of Genetics, Cell Biology, and Development, University of Minnesota, USA

*Corresponding author: Aaron C. Goldstrohm

E-mail: agoldstr@umn.edu


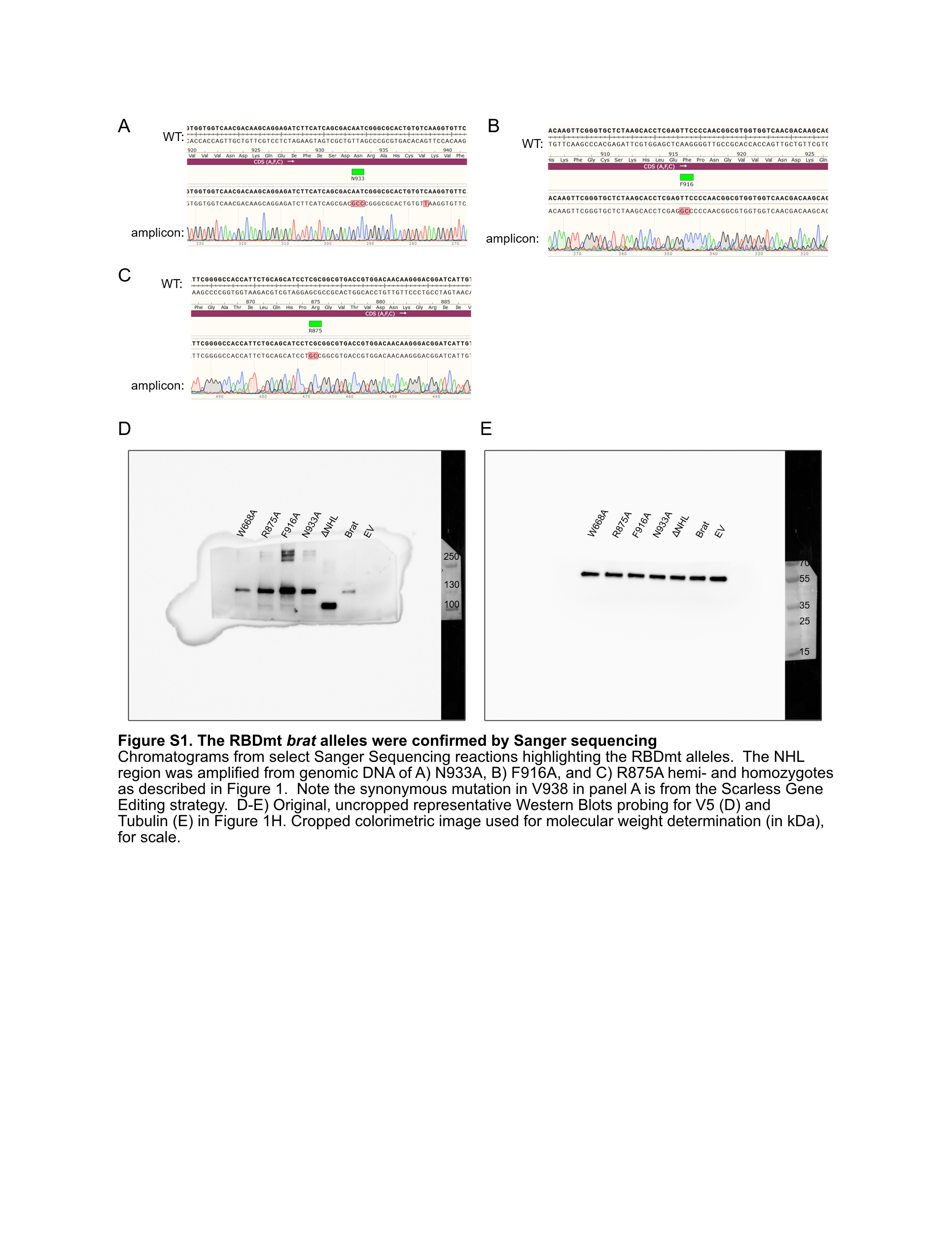


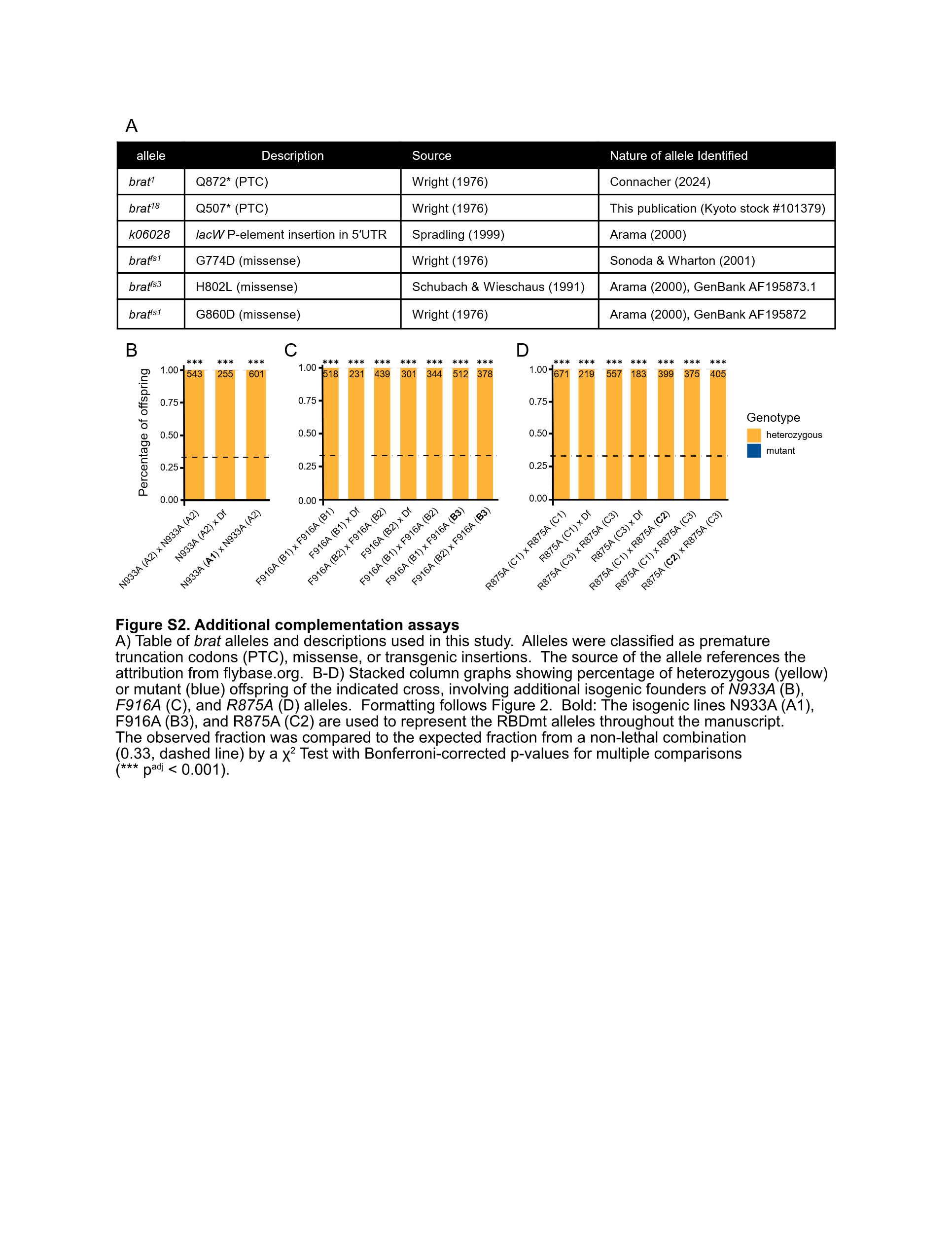


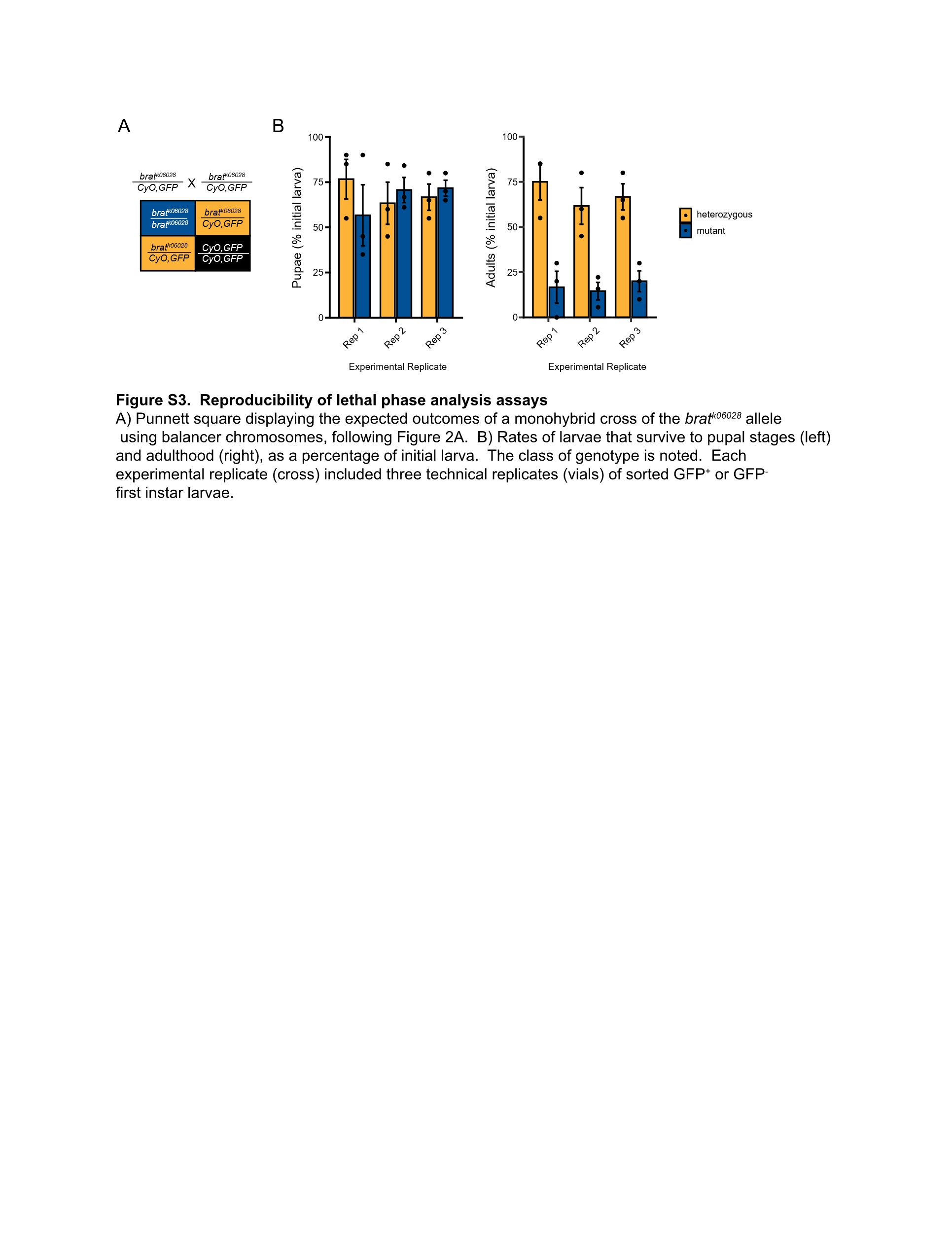


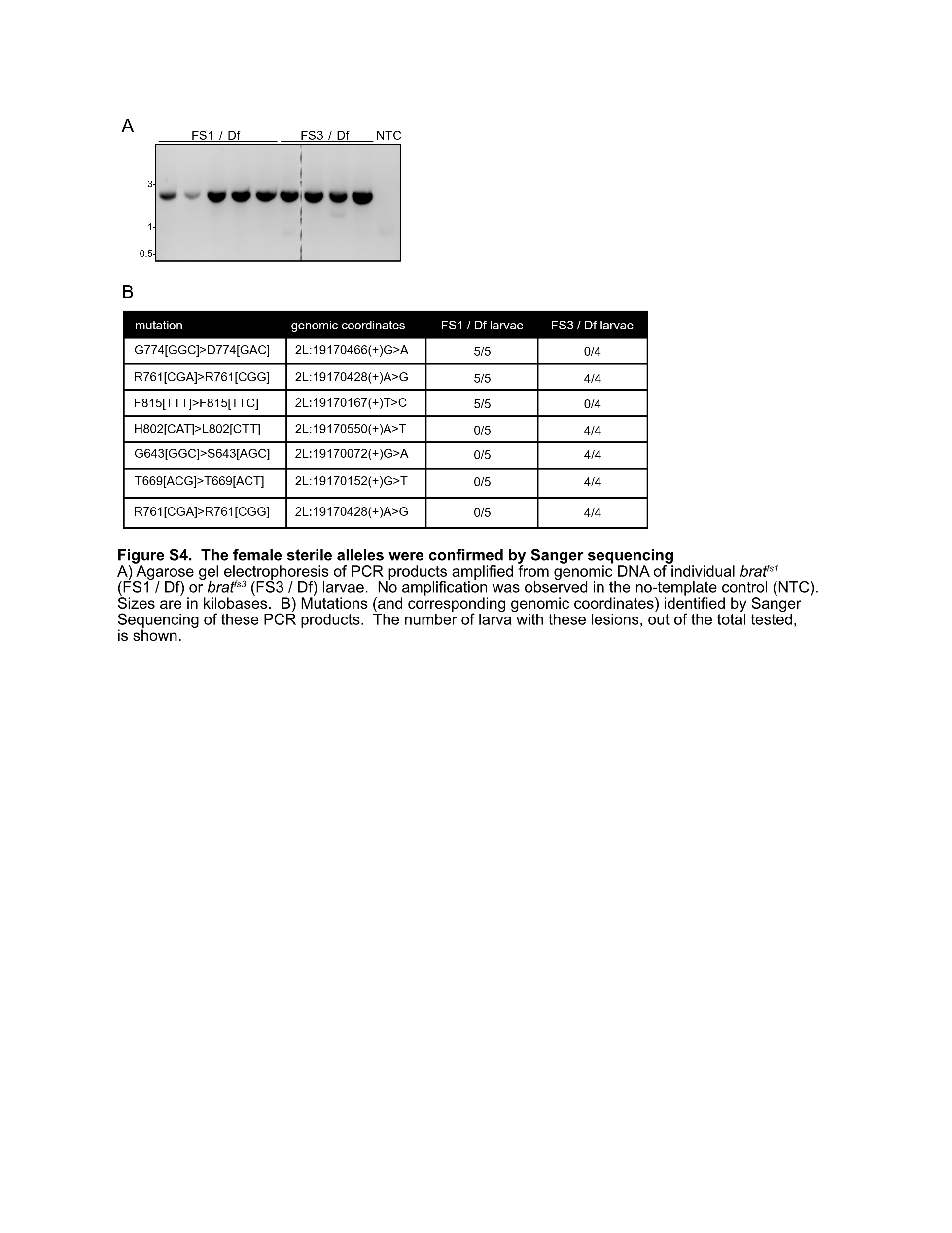

Supplement: Connacher_Combined_Supplementary_Figures_SVG.docx [file KRNB_A_2682069_SM8433.docx]
